# A Whole-Genome Sequencing Association Study of Low Bone Mineral Density Identifies New Susceptibility Loci in the Phase I Qatar Biobank Cohort

**DOI:** 10.3390/jpm11010034

**Published:** 2021-01-07

**Authors:** Nadin Younes, Najeeb Syed, Santosh K. Yadav, Mohammad Haris, Atiyeh M. Abdallah, Marawan Abu-Madi

**Affiliations:** 1 Biomedical Research Center, College of Health Sciences-QU Health, Qatar University, Doha 2713, Qatar; nyounes@qu.edu.qa; 2 Biomedical Informatics Division, Sidra Medicine, Doha 26999, Qatar; nsyed@sidra.org (N.S.); syadav@sidra.org (S.K.Y.); mharis@sidra.org (M.H.); 3 Department of Biomedical Sciences, College of Health Sciences-QU Health, Doha 2713, Qatar; aabdallah@qu.edu.qa; 4 Biomedical and Pharmaceutical Research Unit-QU Health, Qatar University, Doha 2713, Qatar

**Keywords:** bone mineral density, osteoporosis, whole-genome sequencing, genome-wide association, Qatar Biobank, Qatar

## Abstract

Bone density disorders are characterized by a reduction in bone mass density and strength, which lead to an increase in the susceptibility to sudden and unexpected fractures. Despite the serious consequences of low bone mineral density (BMD) and its significant impact on human health, most affected individuals may not know that they have the disease because it is asymptomatic. Therefore, understanding the genetic basis of low BMD and osteoporosis is essential to fully elucidate its pathobiology and devise preventative or therapeutic approaches. Here we sequenced the whole genomes of 3000 individuals from the Qatar Biobank and conducted genome-wide association analyses to identify genetic risk factors associated with low BMD in the Qatari population. Fifteen variants were significantly associated with total body BMD (*p* < 5 × 10^−8^). Of these, five variants had previously been reported by and were directionally consistent with previous genome-wide association study data. Ten variants were new: six intronic variants located at six gene loci (MALAT1/TALAM1, FASLG, LSAMP, SAG, FAM189A2, and LOC101928063) and four intergenic variants. This first such study in Qatar provides a new insight into the genetic architecture of low BMD in the Qatari population. Nevertheless, more studies are needed to validate these findings and to elucidate the functional effects of these variants on low BMD and bone fracture susceptibility.

## 1. Introduction

Bone density disorders are common systemic skeletal conditions characterized by a reduction in bone mass and density that increase the risk of bone fractures [1]. Loss of bone mass can be mild (osteopenia) or severe (osteoporosis). An estimated 200 million people suffer from osteoporosis worldwide, giving rise to ~9 million fractures each year [2]. Loss of bone mass is regarded as a clinically silent condition due to its gradual and asymptomatic nature, making its early diagnosis difficult and often only recognized after the occurrence of the first fracture [3]. The high prevalence of osteopenia and osteoporosis has a significant emotional and financial burden on patients and their families as well as the healthcare systems. Therefore, earlier identification and management of individuals suffering from low bone mineral density (BMD) and osteoporosis may help to curb the impeding societal burden of the disease. BMD serves as a predictor of osteoporotic fractures and is the primary measurement to assess bone health and the gold standard method for this measurement is dual-energy x-ray absorptiometry (iDXA).

Although osteoporosis affects both genders, bone loss in postmenopausal women is accelerated further because of decreasing estrogen levels. In addition, other factors are likely to contribute to bone mass loss; for instance, consumption of tobacco, drinking alcohol, lack of exercise, and low body mass index (BMI) have all been associated with reduced bone mass [4]. Twin- and family-based studies have also suggested a genetic predisposition towards reduced BMD, with heritability estimates of 0.6 to 0.8 [5]. To date, over 518 significant genome-wide loci have been shown to be associated with osteoporosis [6], and a number of meta-analyses have reported a strong relationship between genetic variants and BMD [7,8]. For example, in their meta-analysis of 17 genome-wide association studies (GWAS) of BMD of the femoral neck or lumbar spine, Estrada et al. identified 62 variants showing significant associations [7]. However, determining risk variants and the underlying pathogenetic mechanisms is challenging, and many of the genes that influence BMD are still unknown [9]. The identification of genes that regulate BMD and influence common skeletal fracture sites is critical for understanding the genetic basis of osteoporosis and fracture risk.

According to the International Osteoporosis Foundation, osteoporosis is a neglected disease that is not included in many medical curricula. Unfortunately, the level of awareness about osteoporosis in the Arab countries is poor [10]. In Saudi Arabia, the prevalence of osteopenia and osteoporosis were 46.3% vs 30.7%, and 36.6% vs 34% in men and postmenopausal women, respectively [11]. The only epidemiological study conducted in Qatar on osteoporosis reported a 12.3% prevalence of the disease amongst postmenopausal women [12]. Here we performed whole-genome sequencing association analysis on data generated by the Qatar Biobank (QBB) to identify variants with independent genome-wide significant associations with low BMD in the Qatari population. To our knowledge, this study is the first to investigate genetic risk factors for low BMD in the Qatari population.

## 2. Materials and Methods

### 2.1. Subjects

This was a study of 3000 healthy unrelated Qatari participants in the QBB aged between 18 and 70 years. The details of the QBB are published elsewhere [13]. Briefly, all participants underwent a health examination in the QBB facility at Hamad Medical City. Face-to-face interviews were used to gather information on diet, lifestyle, and sociodemographic information. A specialist nurse interview collected information on general health, use of medications, and family history. Patients with chronic diseases including cancer, diabetes, high cholesterol, Parkinson’s disease, and thyroid diseases were excluded as these diseases might affect the bone mass or metabolism. In addition, related individuals were excluded from the study to minimize the effect of genetic relatedness on association mapping. These exclusions eliminated the interference of known environmental factors and pathological conditions on BMD. All participants provided informed consent, and the QBB provided ethical approval (MOPH-AQBB-000222).

### 2.2. iDXA Scan

Full-body dual-energy iDXA (General Electric, Boston, MA, USA) scans to assess BMD were performed by certified technicians. Seven BMD measurements were obtained: lumbar spine (L1-L4), pelvis, trunk, femoral neck, Ward’s triangle, trochanter, and total body BMD. We obtained the iDXA scan results as absolute BMD values (g/cm^2^) and young adult T-scores. We used the osteoporosis diagnostic criteria of WHO in which T-score ≥ −1 indicates normal BMD, T-score < −1.0 to >−2.0 indicates osteopenia, while T-score ≤ −2.0 indicates osteoporosis [14]. All participants were screened with the same iDXA machine to avoid any variation in BMD measurements.

### 2.3. Genotyping and Bioinformatics Analysis

Blood samples were centrifuged at 3750 rpm for 10 min at 4 °C. DNA was extracted from 5 mL blood using the Puregene DNA Isolation Kit (Gentra Systems, Minneapolis, MN, USA). Quantification was performed using a Qubit 2.0 Fluorometer (Invitrogen, Carlsbad, CA, USA). Whole-genome sequencing was conducted by QBB on DNA samples from 3000 participants as per QBB procedures using Illumina HiSeq X Ten sequencers and converted from native BCL format to paired-end FASTQ format using bcl2fastq. Raw data quality was assessed using fastqc. Data passing quality control were aligned to the reference genome (GRCh37) using bwa-kit6 aligner (v7.12). Variant calling was conducted via GATK7v3.3 HaplotypeCaller and the resulting VCF8 files were annotated using snpeffv4.1b.

We conducted an extensive quality control (QC) assessments to ensure the best possible quality of our data. Samples with any discrepancies in the labeling including females with Y chromosome single nucleotide polymorphism (SNP) genotypes, duplicated, or unknown identification numbers were excluded. In addition, single nucleotide polymorphisms (SNPs) were excluded if the genotyping call rate is less than 95% or heritability error rates greater than 1%. Moreover, SNPs with minor allele frequency (MAF) less than 1% or showing a departure from Hardy–Weinberg equilibrium *p* < 10^−6^ were excluded. Further, SNPs on chromosome 22 were used for an identity-by-descent (IBD) analysis in PLINK to further ensure that we do not have any related individuals. Pairs of individuals with a relatedness measure (pi-hat) value > 0.9 were considered to be indicative of a duplicate sample. A genome-wide significance of *p* < 5 × 10^−8^ was used, in which SNPs reported to be lower than this P-value were considered as significantly associated with low BMD. We conducted all QC analyses using the PLINK 2.0 toolset.

### 2.4. Statistical Analysis

We took into consideration the admixture of the Qatari population. Therefore, the population stratification was corrected by principal component analyses (PCA) using EIGENSTRAT analyses [15]. The four key principal components (PCs) derived from a multi-dimensional scaling analysis of identity-by-state (IBS) distances using PLINK 2.0 software. We adjusted the raw BMD values by considering the first four PCs along with gender, age, weight, BMI, and vitamin D as covariates in the regression analysis. Linear regression analysis was performed using PLINK 2.0 to obtain the regression coefficient and Wald test asymptotic *p*-value. We used the qqman package to generate the quantile-quantile (Q-Q) and Manhattan plots [16]. Annovar was used to determine the chromosomal locations and variant annotations [17]. Missense SNPs were included as potential causal variants if the log of their *p*-value was within 50% of the log of the *p*-value for the sentinel SNP. Regulatory SNPs were identified using data from ENCODE. SNPs meeting genomic significance thresholds were considered novel variants when not previously reported in UK Biobank, Open Target Genetics, Clinvar, GEFOS, GRCh37 Ensembl, gnomAD, and NCBI cohorts and databases. SNPs information data was retrieved from Ensembl, NCBI, GRCh37, and Clinvar.

## 3. Results

### 3.1. Baseline Characteristics

The demographic characteristics of the 3000 included participants are summarized in Table 1. 52% of the participants were female, and 48% were males. The mean age was 36.59 ± 10.6 for men and 36.19 ± 10.6 for women (Table 1). Significant differences were observed in height and weight between males and females (*p* < 0.001). However, there was no significant difference in the BMI (28.29 ± 5.4 and 28.48 ± 6.2 kg/m^2^ in males and females, respectively) (Table 1).

The T-scores for the QBB cohort are presented in Table 2. With respect to total body T-scores, 9.7% of the sample were in the osteopenia and 1.1% were in the osteoporosis WHO categories (Table 2).

BMD values were categorized using the T-scores. Based on the osteoporosis diagnostic criteria of WHO; T-score ≥ −1 indicates normal BMD, T-score < −1.0 to > −2.0 indicates osteopenia, while T-score ≤ −2.0 indicates osteoporosis.

A significant difference in the mean total body BMD T-scores for males and females was observed (0.64 ± 1.16 and 0.17 ± 1.10, respectively) (*p* < 0.01) (Table 3). The T-score measurements and differences were similar to total body T-scores for the femoral trochanter, femoral upper neck, and Ward’s triangle.

### 3.2. Variants Associated with BMD

**Figure 1:**
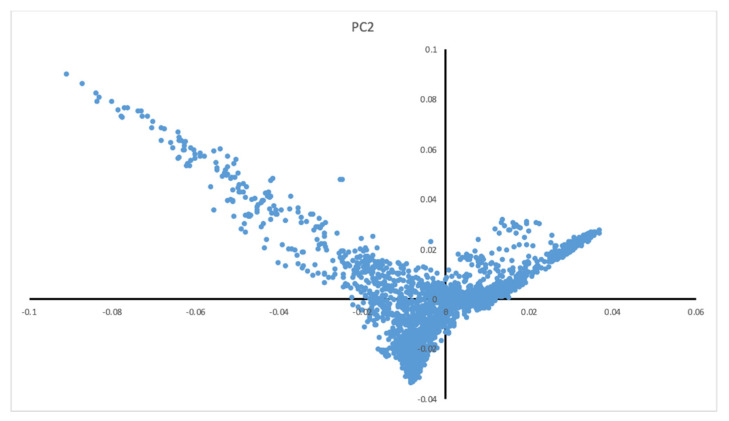
Principle component analysis of the study cohort. The Qatari population can be divided into three main clusters of Arabian, Persian, and African mixed origin.

**Figure 2:**
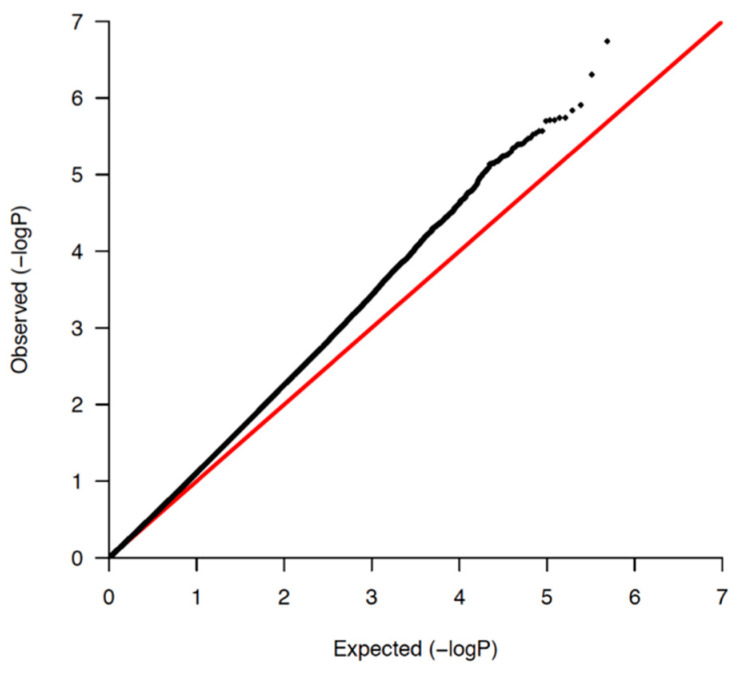
Quantile-quantile plots (QQ-plots) for total body BMD.

In total, 1,084,750 variants passed QC, with a general genotyping rate of 99.93%. According to the PCs, the Qatari population could be divided into three main clusters of Arabian, Persian, and mixed African descent (Figure 1). As depicted in the Q-Q plot (Figure 2 and Figure S1), a deviation from the random distribution for some of the tested SNPs was observed, which indicates that some SNPs were indeed associated with BMD. Nineteen autosomal SNPs were significantly associated with total body, spine, pelvis, trunk, and Ward’s triangle BMD (*p* < 5 × 10^−8^; Table 4 and Table S1).

**Figure 3:**
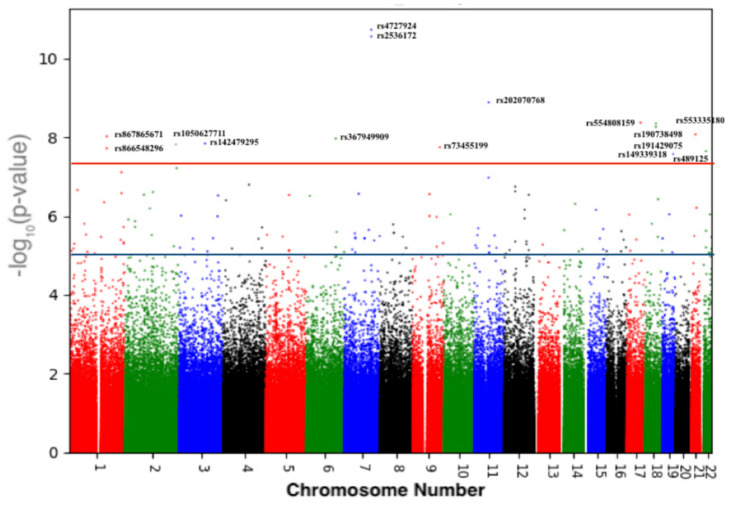
Manhattan plot showing the significantly associated single nucleotide polymorphisms (SNPs) with total body BMD of 3000 Qatari participants. The X-axis shows the genomic position on the 22 chromosomes. The Y-axis shows the negative log-base-10 of the *p* value. The red line shows the genome-wide significance level of 5.0 × 10^−8^.

Figure 3 and Figure S2 show the Manhattan plots. Fifteen variants were associated with low total body BMD, all of which were intronic variants located in different genes except for four intergenic variants (Table 4). The strongest association was for rs202070768 in the long non-coding (lnc)RNA MALAT1 and its antisense TALAM1 on chromosome 11 (*p* = 1.3 × 10^−9^) (Table 4). Four different intronic variants were detected in four different genes with a genome-wide significance of *p* < 5 × 10^−8^: rs867865671 in FASLG on chromosome 1; rs105062771 in SAG on chromosome 2; rs142479295 in LSAMP on chromosome 3; and rs73455199 in FAM189A2 on chromosome 9.

Finally, rs149339318 was within the uncharacterized gene LOC101928063 on chromosome 19 (Table 4). In GTEx eQTL analysis release version 8, rs149339318 is associated with the expression of two genes: the lncRNA RP11-15A1.3 (*p* = 8.6 × 10^−5^) and ZNF404 (zinc finger protein 404) (*p* = 2.2 × 10^−7^). The eQTL effect of re149339318 has been reported in different tissues including artery-tibial, nerve-tibial, and sun-exposed skin.

Four different intergenic variants were found in four different genes with a genome-wide significance of *p* < 5 × 10^−8^: rs554808159 on chromosome 17, rs866548296 on chromosome 1, rs367949909 on chromosome 6, and rs199894228 on chromosome 21 (Table 4).

### 3.3. Validation of Previous Associations with BMD

Out of these fifteen variants associated with total body BMD, five variants were previously reported in the UK-Biobank and GEFOS (Table 4 and Table S1). Two of these intronic variants were also associated with spine and trunk BMD as well as total body BMD: rs4727924 (*p* < 1.8 × 10^−11^) and rs2536172 (*p* < 2.75 × 10^−11^) (Table 4 and Table S1), both of which are on chromosome 7 at q31.31, which contains three genes: WNT16, FAM3C, and C7orf58. Another two previously reported intronic variants (rs190738498 and rs191429075) were in PIGN on chromosome 18, while the final variant rs489125 was in CRYBB2P1 on chromosome 22.

## 4. Discussion

This is the first whole-genome sequencing association study to include seven BMD measurements in a large cohort of Arabic ethnicity. We detected new variants by direct whole genome sequencing, which has increased power and precision for finding causative variants than array and imputation technologies. Furthermore, the Qatari population is known for high consanguinity (~65% first-cousin consanguinity) [18], and consanguineous populations have elevated homozygosity, facilitating the detection of novel genetic variants that cannot be detected in outbred populations [19]. Fifteen SNPs with genome-wide significance with total body BMD were identified, five of which had previously been reported by the UK biobank and GEFOS as well as ten new variants. These data illustrate the complexity of the low BMD phenotype, with several common genomic variants contributing to small phenotypic effects.

Out of the ten new SNPs associated with total body BMD in Qatari participants, six were intronic variants located at six gene loci: MALAT1/TALAM1, FASLG, LSAMP, SAG, FAM189A2, and LOC101928063, while the remaining four were intergenic. rs202070768, an intronic variant, is located at 11q13.1 and overlaps with the lncRNA metastasis-associated lung adenocarcinoma transcript 1 (MALAT1) gene, along with its antisense transcript TALAM1. MALAT1 positively regulates mesenchymal stem cell osteogenic differentiation [20] and a potential therapeutic target in patients with osteolysis associated with knee replacement [21]. A recent meta-analysis reported a strong association between high expression of MALAT1 and unfavorable prognosis of patients suffering from osteosarcoma, making it a potential prognostic biomarker for this cancer [22]. rs1050627711 is an intronic variant located at q37.1 overlapping with the S-antigen visual arrestin (SAG) gene, which encodes arrestin. In mice, animals treated with β-arrestins have been shown to have an increased bone formation rate, osteoblast numbers, and accelerated mineral apposition [23]. The intronic variant rs867865671 located at 1q24.3 overlaps with the Fas ligand (FASLG) gene. The FASL signaling pathways play a critical role in bone development. Estrogen is a major hormonal regulator of bone metabolism and plays a principal role in maintaining the balance between osteogenesis (mediated by osteoblasts) and bone resorption (mediated by osteoclasts) by inducing osteoclast apoptosis. Through an autocrine mechanism, estrogen induces osteoclast apoptosis by inducing FASLG pathway in osteoblasts [24]. rs142479295 is found at 3q13.32, overlapping with the limbic system-associated membrane protein (LSAMP) gene. Copy number alterations at this locus are associated with osteosarcomas [25]. rs73455199 is located at 9q21.12 and overlaps with the FAM189A2 gene, duplications of which have been reported in progressive non-syndromic deafness [26]. rs149339318, a deletion of two base pairs, is found at 19q13.3 overlapping with the uncharacterized LOC101928063 gene. A recent GWAS meta-analysis of 30 array genotyping studies (66,628 participants) found rs7255083, a lead SNP approximately 165 kbp away from rs149339318, to be associated with total body BMD [27]. Interestingly, in the GTEx database, the rs149339318 variant influences the expression of two genes: RP11-15A1.3 and ZNF404, where ZNF404 is significantly overexpressed in sun-exposed skin (but not non-sun-exposed skin) in heterozygous carriers of the rs149339318 polymorphism. This highlights that at least some disease risk may arise from a complex interaction between the genetic risk factors and environmental risk factors implicated in vitamin D activation pathways. Although there is ambiguity about the roles played by the ZNF404 protein in bone metabolism, gene network prediction analysis in osteoblasts using GIANT2.0 web tool (minimum interaction confidence set to 0.47) [28] showed a strong interaction between ZNF404 and the bone morphogenetic proteins BMPR2 and BMPR1B, which may suggest an important role for ZNF404 in bone morphogenesis. However, the exact function of this insertion/deletion SNP rs149339318 in BMD requires further experimental investigation and validation.

We confirmed five genomic variants previously reported in the UK Biobank and GEFOS studies that were also associated with total body BMD in the Qatari population. Two variants, rs4727924 and rs2536172 located at 7q31.31, were associated with total body, spine, and femoral trochanter BMD. This region contains three genes, WNT16, FAM3C, and C7orf58. WNT16 is a biologically attractive candidate for mediating BMD since it belongs to the Wnt protein family, which is known to play an essential role in bone physiology, particularly bone formation and remodeling [29]. FAM3C is widely expressed in different cell types including osteoblasts [30]. To our best knowledge, C7orf58, also called CPED1, has not been associated with any disease and no mutations in this gene have been reported. Two variants replicated in our study and associated with total body BMD, rs190738498 and rs191429075, are located at 18q21.33. PIGN, contained within this region, is one of more than 20 genes associated with glycosylphosphatidylinositol anchor biosynthesis. Recently, the PIGN mutation has been identified in an Israeli Arab family with multiple congenital anomalies, which include defects in the heart and skeleton [31]. However, the exact mechanism leading to these phenotypes is unknown. Finally, the last replicated variant reported in our study was rs489125 located at 22q12.1 overlapping with CRYBB2P1. The CRYBB2P1 is a pseudogene belonging to the β-crystallin family and is transcribed in almost all tissues except eye tissues. No proteins have yet been associated with CRYBB2P1, and there is currently no clear mechanism of how this gene affects BMD level.

Our study has two main limitations. First, the moderate sample size in the discovery phase might not have had optimal statistical power. Whole genome sequencing association studies require large populations to detect variants with small effect sizes and a higher statistical significance threshold [32]. In phase II study, a larger sample size will be included. This will allow us to increase the study power and perform association analyses between the three main cluster descents (Arabian, Persian, and mixed African) of the Qatari population. Second, our cohort was limited to the Qatari population, which although useful for discovery, may not be generalizable. Although PC analysis showed that the analyzed Qatari population consisted of three distinct genetic descents (Arabian, Persian, and mixed African), providing some diversity, the findings from this study need confirming in different populations.

## 5. Conclusions

This is the first whole-genome sequencing association study of low BMD in an Arab country. The ten new genetic variants described here are of major importance not only for the local population but also for the wider study of the molecular mechanisms controlling bone remodeling and development. This study highlights a new variant associated with low BMD in MALAT1 in which its expression was previously associated with mesenchymal stem cell osteogenic differentiation. Another interesting discovery was the two base pair insertion/deletion rs149339318 variant, which might influence ZNF404 under specific environmental conditions and in turn effect vitamin D metabolism and bone morphogenetic proteins. Knowledge of population-specific variants will be useful for understanding disease pathogenesis and designing polygenic risk scores for clinical application. Our results may help to identify individuals at high risk of osteopenia and osteoporosis. Individuals presented with such risk should be screened for BMD using iDXA.

## Figures and Tables

**Table 1:** The demographics of the study cohort.

Parameter	Male (±SD)	Female (±SD)
Number of participants	1442	1558
Age (years)	36.59 (±10.6)	36.19 (±11.6)
Height (cm)	172.37 (±6.3)	158.09 (±5.9)
Weight (kg)	84.84 (±17.9)	71.18 (±15.9)
BMI	28.29 (±5.4)	28.48 (±6.2)

**Table 2:** Summary of bone mineral density (BMD) T-score measurements.

	Normal	Osteopenia	Osteoporosis
Total body	2617 (89%)	285 (9.71%)	33 (1.1%)
Femoral Trochanter	1991 (68.6%)	718 (24.7%)	194 (6.68%)
Femoral upper neck	2291 (78%)	496 (16.9%)	116 (3.95%)
Ward’s triangle	1643 (56.5%)	885 (30.5%)	375 (12.9%)

**Table 3:** Summary of BMD T-score measurements between males and females.

	Total Cohort Mean (SD)	Male Mean (SD)	Female Mean (SD)	*p*-Value
Total body	0.39 (±1.15)	0.64 (±1.16)	0.17 (±1.10)	*p* < 0.001
Femoral trochanter	−0.37 (±1.177)	−0.59 (±1.25)	−0.67 (±1.01)	*p* < 0.001
Femoral upper neck	0.06 (±1.31)	0.37 (±1.427)	−2.25 (±1.11)	*p* < 0.001
Ward’s triangle	−0.69 (±1.22)	−0.47 (±1.33)	−0.89 (±1.08)	*p* < 0.001

**Table 4:** Genome-wide significant total body bone mineral density variants.

Chr	RefSNP	Position (GRCh37)	Band	Ancestor Allele	Effect Allele	*p*-Value	Gene
**Replicated in the UK-Biobank and GEFOS studies**							
**7**	rs4727924	121031879	q31.31	C	T	1.86 × 10^−11^	*FAM3C*
7	rs2536172	120997560	q31.31	A	T	5.75 × 10^−8^	*FAM3C/WNT16* intronic
18	rs190738498	59831463	q21.33	G	A	5.71 × 10^−8^	*PIGN*: Intronic
18	rs191429075	59790212	q21.33	C	T	6.93 × 10^−8^	*PIGN*: Intronic
22	rs489125	25911056	q12.1	G	A	2.25 × 10^−8^	*CRYBB2P1*: intronic
**New findings in the Qatari population**							
11	rs202070768	65273453	q13.1	T	C	1.30 × 10^−9^	*MALAT1/TALAM1*
17	rs554808159	61978607	q23.3	C	T	4.25 × 10^−9^	intergenic
1	rs867865671	172626211	q24.3	A	G	3.03 × 10^−8^	*FASLG*: intronic
1	rs866548296	234651783	q42.2	C	T	7.77 × 10^−8^	intergenic
2	rs1050627711	233310901	q37.1	C	T	6.08 × 10^−8^	*SAG*: Intronic
3	rs142479295	117374777	q13.32	T	Dup T	9.68 × 10^−8^	*LSAMP*: intronic
6	rs367949909	132861904	q23.2	T	C	4.94 × 10^−8^	intergenic
9	rs73455199	71961260	q21.12	A	G	3.38 × 10^−8^	*FAM189A2*: Intronic
19	rs149339318	44503670	q13.31	TA	Del TA	8.35 × 10^−8^	*LOC101928063*: intronic
21	rs199894228/rs553335180	26574354	q21.2	T	Del T	8.44 × 10^−9^	intergenic

## Data Availability

Restrictions apply to the availability of these data. Data was obtained from Qatar Biobank (https://www.qatarbiobank.org.qa/) and are available from Qatar Biobank upon request.

## Supplementary Materials

Supplemental data are available online at https://www.mdpi.com/2075-4426/11/1/34/s1. Table S1: Genome-wide significant total body bone mineral density variants. Lumbar spine, Pelvis, Trunk, and Femoral Ward. Figure S1: quantile-quantile plots (QQ-plots) for Spine, Pelvis, Trunk, Femoral (neck, war, troch) BMD, and Figure S2: Manhattan plots representing genome-wide association results for Spine, Pelvis, Ward’s triangle, and trunk bone mineral density (BMD) of 3000 participants.
