# Supplementary material for: A Whole-Genome Sequencing Association Study of Low Bone Mineral Density Identifies New Susceptibility Loci in the Phase I Qatar Biobank Cohort
Source: J Pers Med. 2021 Jan 7;11(1):34. doi: 10.3390/jpm11010034 (PMC7825795; doi:10.3390/jpm11010034)
Supplement: Supplementary file 1 [file jpm-11-00034-s001.pdf]

Table S1. Genome-wide significant total body bone mineral density variants. Lumber spine, Pelvis, Trunk and Femoral Ward.

**BMD of the Spine**											
**7**	rs4727924	121031879	q31.31	C	T	1.63x10^{-08}	FAM3C/WNT16 intronic	BMD, Fracture risk	UK BioBanK, GEFOS	5.56x10^{-160}, 4.39x10^{-10}	194,39, 335,587
**7**	rs2536172	120997560	q31.31	A	T	5.75x10^{-08}	FAM3C/WNT16 intronic	Fractured /broken bones	UK BioBanK	2.09x10^{-180}	194,398
**BMD of the Pelvis**											
**7**	rs1839588	37979888	p14.1	C	T	2.00x10^{-08}	SFRP4/ intronic	BMD	UK BioBanK	2.70x10^{-24}	194,398
**BMD of the Trunk**											
**7**	rs4727924	121031879	q31.31	C	T	1.63x10^{-08}	FAM3C/WNT16 intronic	BMD, Fracture risk	UK BioBanK,GEFOS	5.56x10^{-160}, 4.39x10^{-10}	194,39, 335,587
**7**	rs2536172	120997560	q31.31	A	T	5.75x10^{-08}	FAM3C/WNT16 intronic	Fractured /broken bones	UK BioBanK	2.09x10^{-180}	194,398
**4**	rs1050715238	95757373	q22.3	A	G	4.77x10^{-08}	BMPR1B/ Intronic	No Data	N/A	N/A	N/A
**11**	rs371319602	96953896	q21	A	C	6.00x10^{-08}	intergenic	No Data	N/A	N/A	N/A
**BMD of the Femoral Ward**											
**2**	rs62150773	109035597	q12.3	G	C	1.72x10^{-08}	intergenic variant	No Data	N/A	N/A	N/A

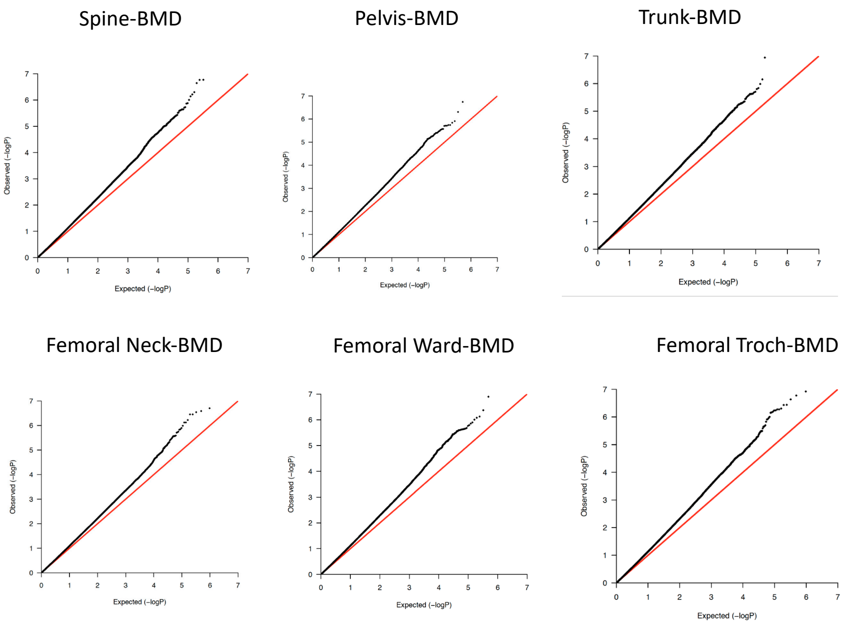

Figure S1. quantile-quantile plots (QQ-plots) for Spine, Pelvis, Trunk, Femoral (neck, war, troch) BMD.

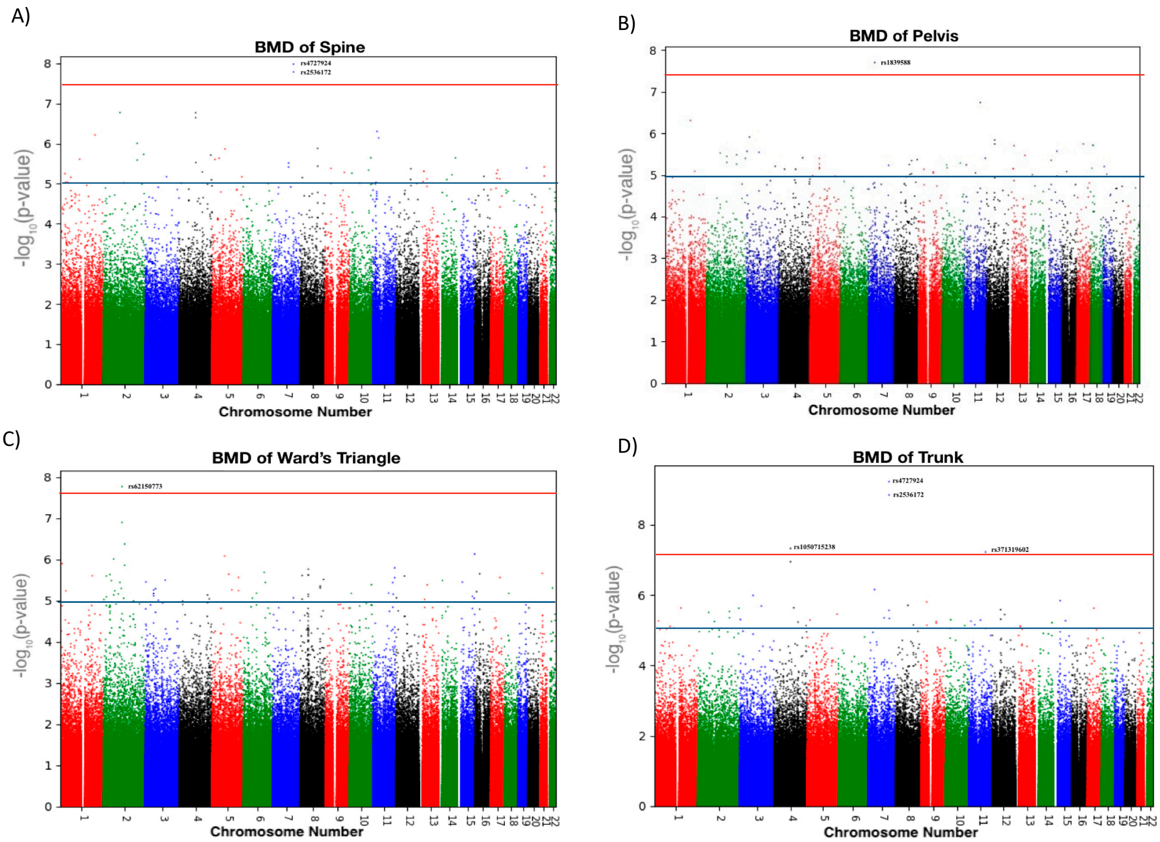

Figure S2. Manhattan plots representing genome-wide association results for Spine, Pelvis, Ward's triangle and trunk bone mineral density (BMD) of 3000 participants. The P values (−log10) are plotted against their respective positions on each chromosome. Results are shown for all variants with significance level $P \leq 10^{-8}$.
